# A classic case suggestive of an unruptured ectopic pregnancy with unexpected finding of a unilateral dermoid cyst intraoperatively: A case report

**DOI:** 10.4102/safp.v62i1.5164

**Published:** 2020-12-09

**Authors:** Matthew A. Benedict, Anthonio O. Adefuye

**Affiliations:** 1 Department of Family Medicine, Faculty of Health Sciences, University of the Free State, Bloemfontein, South Africa; 2 Division of Health Sciences Education, Faculty of Health Sciences, University of the Free State, Bloemfontein, South Africa

**Keywords:** un-ruptured ectopic pregnancy, matured cystic teratoma, general practice, primary healthcare, unilateral dermoid cyst

## Abstract

Acute lower abdominal pain or pelvic pain is a common presenting complaint in women of reproductive age, which can be accounted for by numerous aetiologies. The presentation of lower abdominal pain with associated findings of an adnexal mass on transvaginal ultrasonography and positive beta-human chorionic gonadotropin (β-hCG) (serum and urine) in a sexually active woman of reproductive age is an ectopic pregnancy until proven otherwise. Here, we present a classic case suggestive of an unruptured ectopic pregnancy, with an unexpected finding of a unilateral dermoid cyst intraoperatively in a 33-year-old woman. Findings presented herein suggest that practitioners in our local setting should evaluate patients carefully, and consider neoplasms as a possible source of β-hCG production in sexually active women of reproductive age who present with subacute lower abdominal pain, identified adnexal mass on ultrasonography and positive serum or urine β-hCG readings.

## Introduction

Acute lower abdominal pain or pelvic pain is a common presenting complaint in women of reproductive age, which can be accounted for by numerous aetiologies.^1^ It is, therefore, vital that the attending general practitioner or family physician quickly ascertain the need for acute surgical intervention for conditions that could potentially become life threatening. Chief amongst these aetiologies include the occurrence of ectopic pregnancy (EP) and the presence of an adnexal mass.^1,2^ Ectopic pregnancy refers to the implantation of a fertilised ovum outside the uterine cavity, of which the majority are located in the fallopian tubes;^3^ other locations include ovaries and the abdominal cavity. The estimated global incidence of EP is 1% – 2% of pregnancies,^4^ and it is considered to be one of the leading causes of maternal morbidity and mortality.^5^ In South Africa, EP is reported to occur in 11 of every 1000 reported pregnancies, with an estimated mortality rate of 2%.^6^ Ectopic pregnancy is the prime differential diagnosis in any woman of childbearing age presenting with positive beta-human chorionic gonadotropin (β-hCG) and an adnexal mass. However, it has been reported that certain neoplasms (benign and malignant) can present with similar symptoms.^7^

Mature cystic teratoma (MCT) of the ovary or dermoid cyst is the most common (90%) germ cell tumour of the ovary,^8,9^ accounting for up to 70% of benign ovarian masses during reproductive years, and 20% in postmenopausal women.^10,11^ Mature cystic teratoma is usually asymptomatic until it reaches a considerable size, and it manifests as an adnexal mass associated with abdominal heaviness, dull pain or acute abdominal pain (if the tumour undergoes torsion or rupture).^12^ Here, we present a classic case suggestive of an EP, but with the unexpected finding of a unilateral dermoid cyst intraoperatively, in a 33-year-old woman.

## Case study

A 33-year-old woman presented at the emergency department of a district hospital at 18:35 with a 4-day history of lower abdominal pain radiating to the lower back. She reported no vomiting or any change in bowel habits. According to the patient, she had prior episodes of abnormal vaginal bleeding, which has since stopped. Her last normal menstrual period was 17 March 2018, which corresponds to a gestation of 7 weeks and 2 days; the patient had no history of prior or present use of any form of contraceptive. Her last confinement had been 5 years ago, and had culminated in an uneventful, normal vaginal delivery. No abnormal findings were made from the review of systems and the rest of the history.

**FIGURE 1:**
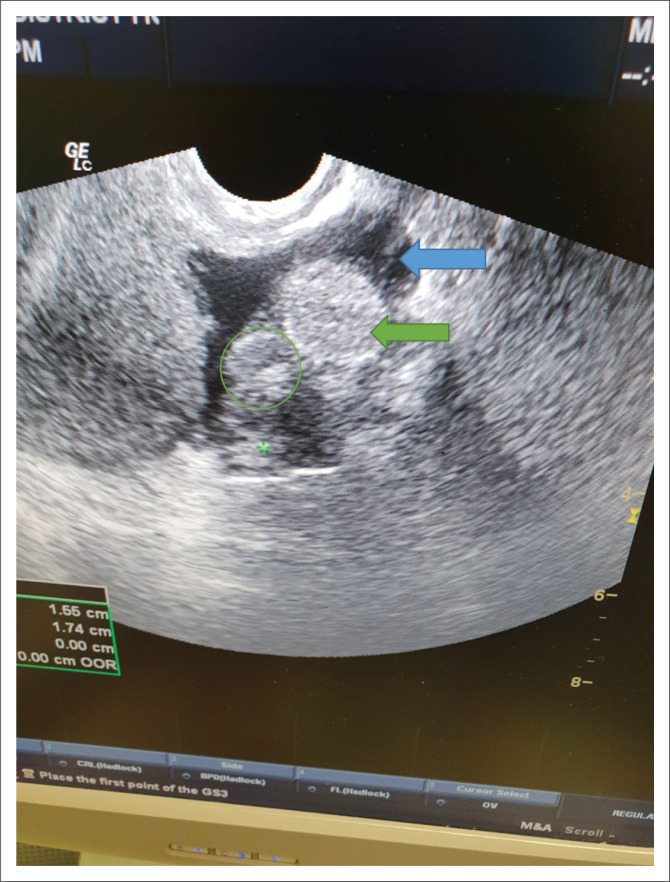
Transvaginal ultrasound showing right adnexal mass (green circle). Note the empty uterus (green arrow) and bladder (blue arrow).

On examination, the patient was slightly anxious, with moderate discomfort resulting from her abdominal pain. She was well hydrated and not pale. Her vital signs were normal (blood pressure 107/73 mmHg, pulse rate 80 beats per minute). Abdominal examination revealed right iliac fossa tenderness with guarding, and no rebound tenderness. No palpable mass or organs were present. Bowel sounds were normal. Vaginal examination revealed a closed, firm cervix without any palpable abnormality. Cervical excitation tenderness was present. A light-brown discharge was observed on the glove. No bleeding was observed. The rest of the systemic examination was normal. A bedside urine β-hCG test that was performed was positive and the transvaginal ultrasonography (performed by the attending medical officer) revealed an empty uterus with a right ‘adnexal mass’ (Figure 1). No fluid was visualised in the pouch of Douglas.

**FIGURE 2:**
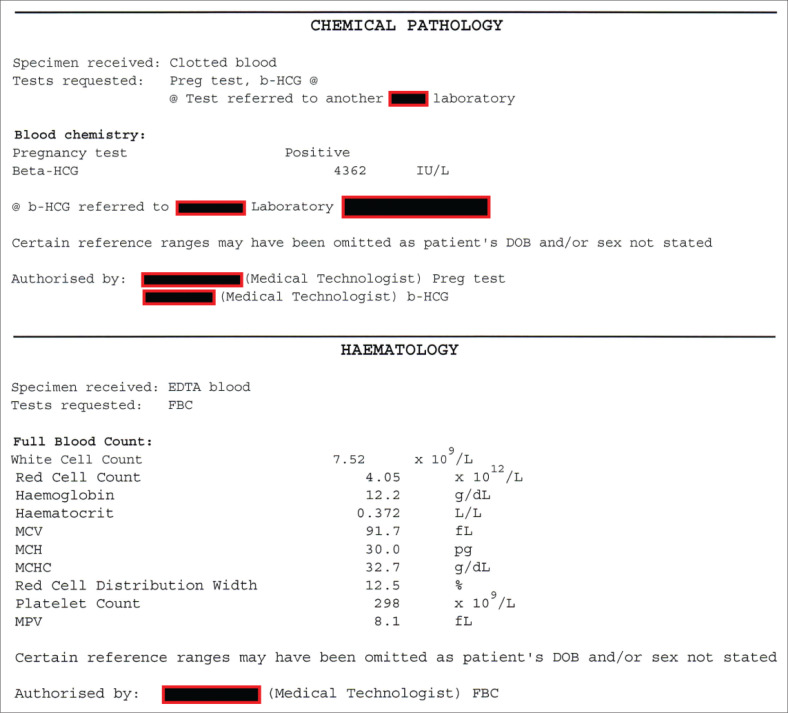
Laboratory report.

Based on these findings, an initial assessment of unruptured EP was made. The patient was kept in the emergency department for close monitoring. At about 2 h post initial assessment (20:35), the patient’s condition was the same, with normal vital signs. Received laboratory reports revealed a haemoglobin level of 12.2 g/dL and a positive serum β-hCG, with a value of 4362 IU/L (within the range expected for patient’s last normal menstrual period) (Figure 2). Patient was kept in the emergency department overnight and she was reassessed the following morning by a more senior and experienced physician (family physician). Ultrasonography was repeated by the hospital sonographer and the findings did not differ from the initial report given by the attending medical officer. Taking the above findings together, a working diagnosis of unruptured EP was made and the patient was booked for an exploratory laparotomy.

**FIGURE 3:**
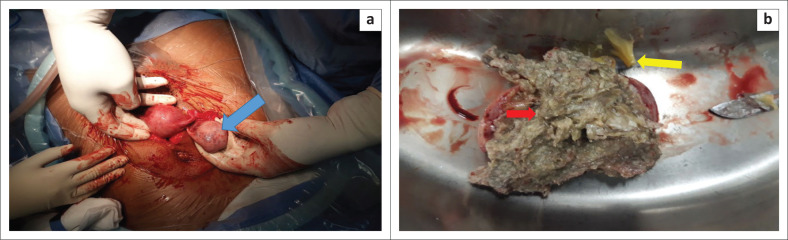
(a) An enlarged right ovary (blue arrow); (b) cut section through the excised ovary. Note the presence of sebum material (yellow arrow) and hair (red arrow).

**FIGURE 4:**
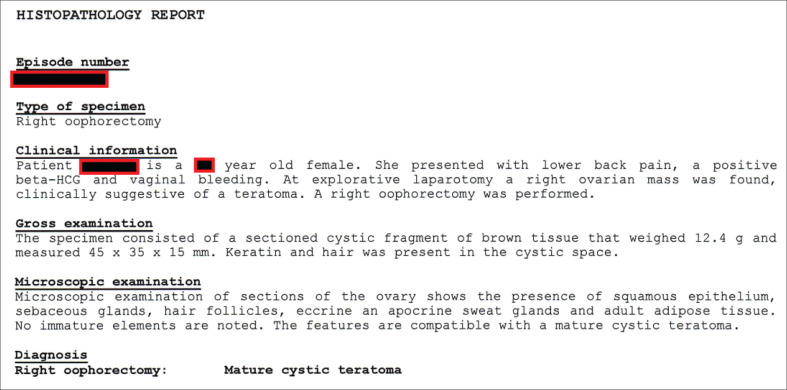
Histopathology report.

Intraoperatively, a right ovarian mass, approximately 7 cm × 5 cm, was found (Figure 3a). Both fallopian tubes and the left ovary were normal. No other abnormality was found. A decision was made on right oophorectomy because of the risk of torsion (a possible cause of the patient’s lower abdominal pain). No EP was found. A cut section through the excised ovary revealed sebum material and hair, suggestive of macroscopic features of cystic teratoma (Figure 3b); the received histopathology report confirmed an MCT (Figure 4).

Post-operatively, the patient’s recovery was uneventful, and she was discharged after 3 days. Her follow-up visit 2 weeks later was also uneventful.

### Ethical consideration

Permission to conduct the research was obtained from the head of the health facility and a written consent was obtained from the patient.

## Discussion

The presentation of lower abdominal pain with associated findings of an adnexal mass on transvaginal ultrasonography and positive β-hCG (serum and urine) in a sexually active woman of reproductive age is an EP until proven otherwise.^13^ The presence of the classical signs and symptoms suggestive of EP, thus, justify the pre-operative diagnosis of non-ruptured EP made in this index case. Similarly, a diagnostic exploratory laparotomy with a high suspicion for unruptured EP is reasonable, given the potential for fatality and as 50% of all ectopic pregnancies are asymptomatic.^14^ However, it is important for practitioners to be aware that positive β-hCG (serum and urine) can be observed in other malignant and non-malignant conditions.^13^

Mature cystic teratoma is the most common neoplasm of the ovary and it is composed of well-differentiated derivations from at least two of the three germ cell layers (ectoderm, mesoderm and endoderm).^15^ It affects a younger age group (mean patient age of 30 years),^16^ which corresponds with the age of the patient in the index case (33 years). The unilateral occurrence of MCT on the right ovary, as seen in the index case, corroborates prior findings that state that MCTs are mostly unilateral and occur more frequently on the right side (72.2%).^17^ Whilst most MCTs can be diagnosed with ultrasound, the ultrasound diagnosis is complicated by the fact that these tumours may have a variety of appearances.^16^ The three main pathognomonic ultrasound findings of MCT include a cystic lesion with a dense echogenic tubercle (Rokitansky nodule) projecting into the cyst lumen;^18^ a diffuse or partially echogenic mass with the echogenic area usually demonstrating sound attenuation owing to sebaceous material and hair within the cyst cavity;^19,20^ and multiple thin, echogenic bands caused by hair in the cyst cavity.^16^ The diagnosis of MCT on computed tomography and magnetic resonance imaging is fairly straightforward,^21^ however, these facilities are lacking in most rural district hospitals in South Africa and, indeed, Africa. In resource-poor settings, serial serum β-hCG assay can be used as a pointer to prompt the consideration of other differential diagnoses to EP, as opposed to doing a single assay, as in the index case. If β-hCG levels increase by less than 50% during a 48-h period, there is usually a non-viable pregnancy associated, whether intra- or extra-uterine.^22,23^ Ectopic pregnancy should be suspected when a β-hCG plateau is reached early in the pregnancy (i.e. before 9–11 weeks).^23^ Mature cystic teratoma can be associated with complications from rupture, malignant degeneration or (most commonly) torsion.^16^

In conclusion, careful evaluation of patients and consideration of neoplasms as a source of β-hCG production in the presence of subacute lower abdominal pain, and identified adnexal mass on ultrasound in sexually active women of reproductive age is essential in our local setting.

## References

[1] Johnson N, Gist W, Akinlaja O, Gist B. Bilateral ovarian teratomas with concurrent ectopic pregnancy at diagnostic laparoscopy. Austin J Womens Health. 2014;1(1):2.

[2] Guidelines in Practice. Pelvic pain in women: What’s the diagnosis? [homepage on the Internet]. [2020 April 06]. Available from: https://www.guidelinesinpractice.co.uk/womens-health/pelvic-pain-in-women-whats-the-diagnosis/453747.article

[3] Crochet JR, Bastian LA, Chireau MV. Does this woman have an ectopic pregnancy? The rational clinical examination systematic review. JAMA. 2013;309(16):1722–1729. 10.1001/jama.2013.3914 23613077

[4] Barnhart KT. Ectopic pregnancy. N Engl J Med. 2009;361(4):379–387. 10.1056/NEJMcp0810384 19625718

[5] Timor-Tritsch IE, Monteagudo A, Mandeville EO, Peisner DB, Anaya GP, Pirrone EC. Successful management of viable cervical pregnancy by local injection of methotrexate guided by transvaginal ultrasonography. Am J Obstet Gynecol. 1994;170(3):737–739. 10.1016/S0002-9378(94)70273-X 8141192

[6] Nzaumvila DK, Govender I, Ogunbanjo GA. An audit of the management of ectopic pregnancies in a district hospital, Gauteng, South Africa. Afr J Prim Health Care Fam. 2018;10(1):1–8. 10.4102/phcfm.v10i1.1757 PMC6244319 30456972

[7] Chew KT, Abu MA, Ahmad MF, Abdul Ghani NA. Bilateral mature cystic teratoma masquerading as an ectopic pregnancy: A case report and review of the literature. J Gynecol Surg. 2018;34(5):252–254. 10.1089/gyn.2018.0022

[8] Sinha A, Ewies AA. Ovarian Mature Cystic Teratoma: Challenges of surgical management. Obstetrics and Gynecology International. 2016;2016:1–7. 10.1155/2016/2390178 PMC4823513 27110246

[9] Mori Y, Nishii H, Takabe K, et al. Preoperative diagnosis of malignant transformation arising from mature cystic teratoma of the ovary. Gynecol Oncol. 2003;90(2):338–341. 10.1016/S0090-8258(03)00259-2 12893196

[10] Shalev E, Bustan M, Romano S, Goldberg Y, Ben-Shlomo I. Laparoscopic resection of ovarian benign cystic teratomas: Experience with 84 cases. Hum Reprod. 1998;13(7):1810–1812. 10.1093/humrep/13.7.1810 9740429

[11] Canis M, Mage G, Pouly JL, Wattiez A, Manhes H, Bruhat MA. Laparoscopic diagnosis of adnexal cystic masses: A 12-year experience with long-term follow-up. Obstet Gynecol. 1994;83(5 Pt 1):707–712. 8164928

[12] Sait K, Simpson C. Ovarian teratoma diagnosis and management: Case presentations. J Obstet Gynaecol Can. 2004;26(2):137–142. 10.1016/S1701-2163(16)30489-3 14965479

[13] Kucera C, Cox-Bauer C, Miller C. Apparent ectopic pregnancy with unexpected finding of a germ cell tumor: A case report. Gynecol Oncol Rep. 2017;21:31. 10.1016/j.gore.2017.05.004 28664181 PMC5479954

[14] American College of Obstetricians Gynecologists. ACOG Practice Bulletin No. 94: Medical management of ectopic pregnancy. Obstet Gynecol. 2008;111(6):1479. 10.1097/AOG.0b013e31817d201e 18515537

[15] Sahin H, Abdullazade S, Sanci M. Mature cystic teratoma of the ovary: A cutting edge overview on imaging features. Insights Imaging. 2017;8(2):227–241. 10.1007/s13244-016-0539-9 28105559 PMC5359144

[16] Outwater EK, Siegelman ES, Hunt JL. Ovarian teratomas: Tumor types and imaging characteristics. Radiographics. 2001;21(2):475–490. 10.1148/radiographics.21.2.g01mr09475 11259710

[17] Ismail SR. An evaluation of the incidence of right-sided ovarian cystic teratoma visualized on sonograms. J Diagn Med Sonogr. 2005;21(4):336–342. 10.1177/8756479305279035

[18] Quinn S, Erickson S, Black W. Cystic ovarian teratomas: The sonographic appearance of the dermoid plug. Radiology. 1985;155(2):477–478. 10.1148/radiology.155.2.3885313 3885313

[19] Patel MD, Feldstein VA, Lipson SD, Chen DC, Filly RA. Cystic teratomas of the ovary: Diagnostic value of sonography. AJR Am J Roentgenol. 1998;171(4):1061–1065. 10.2214/ajr.171.4.9762997 9762997

[20] Dodd GD 3rd, Budzik R Jr. Lipomatous tumors of the pelvis in women: Spectrum of imaging findings. AJR Am J Roentgenol. 1990;155(2):317–322. 10.2214/ajr.155.2.2115259 2115259

[21] Guerriero S, Mallarini G, Ajossa S, et al. Transvaginal ultrasound and computed tomography combined with clinical parameters and CA-125 determinations in the differential diagnosis of persistent ovarian cysts in premenopausal women. Ultrasound Obstet Gynecol. 1997;9(5):339–343. 10.1046/j.1469-0705.1997.09050339.x 9201878

[22] Lin EP, Bhatt S, Dogra VS. Diagnostic clues to ectopic pregnancy. Radiographics. 2008;28(6):1661–1671. 10.1148/rg.286085506 18936028

[23] Lipscomb GH, Stovall TG, Ling FW. Nonsurgical treatment of ectopic pregnancy. N Engl J Med. 2000;343(18):1325–1329. 10.1056/NEJM200011023431807 11058678

